# Lockdown impacts on residential electricity demand in India: A data-driven and non-intrusive load monitoring study using Gaussian mixture models

**DOI:** 10.1016/j.enpol.2022.112886

**Published:** 2022-05

**Authors:** Ramit Debnath, Ronita Bardhan, Ashwin Misra, Tianzhen Hong, Vida Rozite, Michael H. Ramage

**Affiliations:** a Energy Policy Research Group, Cambridge Judge Business School, University of Cambridge, Cambridge, CB2 1AG, UK; b Centre for Natural Material Innovation, Department of Architecture, University of Cambridge, Cambridge, CB2 1PX, UK; c Division of Humanities and Social Science, California Institute of Technology, Pasadena, CA, 91125, USA; d Sustainable Design Group, Department of Architecture, University of Cambridge, Cambridge, CB2 1PX, UK; e The Robotics Institute, Carnegie Mellon University, Pittsburgh, PA, 15213-3890, USA; f Building Technology and Urban Systems Division, Lawrence Berkeley National Laboratory, Berkeley, CA, 94720, USA; g Energy Efficiency Division, International Energy Agency, Paris, 75015, France

**Keywords:** COVID-19, Work-from-home, NILM, Machine learning, Mixture models, India, AI, Artificial Intelligence, GMM, Gaussian Mixture Models, NEEM, National Energy End-use Monitoring, WFH, Work-from-Home, NILM, Non-intrusive Load Monitoring, BR1, 1-bedroomunit, BR2, 2-bedroom unit, BR3, 3-bedroom unit, M3BR, More than 3-bedroom unit, RM1, 1-room unit, HDD, Heating Degree Day, CDD, Cooling Degree Day, MDS, Multidimensional Scaling, EM, Expectation–Maximisation algorithm

## Abstract

This study evaluates the effect of complete nationwide lockdown in 2020 on residential electricity demand across 13 Indian cities and the role of digitalisation using a public smart meter dataset. We undertake a data-driven approach to explore the energy impacts of work-from-home norms across five dwelling typologies. Our methodology includes climate correction, dimensionality reduction and machine learning-based clustering using Gaussian Mixture Models of daily load curves. Results show that during the lockdown, maximum daily peak demand increased by 150–200% as compared to 2018 and 2019 levels for one room-units (RM1), one bedroom-units (BR1) and two bedroom-units (BR2) which are typical for low- and middle-income families. While the upper-middle- and higher-income dwelling units (i.e., three (3BR) and more-than-three bedroom-units (M3BR)) saw night-time demand rise by almost 44% in 2020, as compared to 2018 and 2019 levels. Our results also showed that new peak demand emerged for the lockdown period for RM1, BR1 and BR2 dwelling typologies. We found that the lack of supporting socioeconomic and climatic data can restrict a comprehensive analysis of demand shocks using similar public datasets, which informed policy implications for India's digitalisation. We further emphasised improving the data quality and reliability for effective data-centric policymaking.

## Highlights

- Lockdown caused shifts in daily peak demand across residential units in 13 cities in India
- Daily peak demand increase by 150–200% due to lockdown in households with one or two rooms
- Work-from-home effects identified through emerging peak demand across typologies
- Demonstrated the need for robust public data quality as digitalisation policy for India

## 1 Introduction

Digitalisation will play a vital role in decarbonising building energy systems while improving their sustainability and operational efficiency. Recent advances in digitalisation in building energy systems fuelled by artificial intelligence (AI) has paved new ways for fast and cost-effective data acquisition and advanced analytics. For example, non-intrusive load monitoring (NILM) is a popular approach to estimate appliance-level electricity consumption from cumulative consumption data of households (Beckel et al., 2014). Although the current NILM literature focuses mainly on algorithm and sensor development, examples of building energy management and policy design applications remain limited (Antonio Ruano et al., 2019). This study uses NILM data from smart meters in Indian households across 13 cities in five climatic zones for estimating the impact of COVID-19 reactive public policy measures like lockdowns on residential electricity demand.

The use of NILM data for evaluating lockdown related demand shift at the household level is novel and timely to understand the effects of the COVID-19 pandemic in the energy sector. India has a unique occupancy characteristic for middle-income urban households. At least one family member will stay indoors during a typical workday and is never wholly empty (09:00 to 17:00 h) (Bardhan and Debnath, 2016). However, with pandemic induced work-from-home (WFH) norms, the structural shifts in residential demand are not known and remains a critical knowledge gap for post-pandemic recovery scenarios. It calls for effective policy actions to save residential consumers from demand imbalance shocks that directly affect the household economy. For example, consumers claimed they were billed three to 15 times the usual levels, with bills for June 2020 reaching above INR 30,000 (∼USD 403) (Anwesha Madhukalya, 2020; Trends Desk, 2020).

India went on a complete nationwide lockdown between March 25, 2020 to May 31, 2020 as a reactive measure to contain the COVID-19 pandemic (Debnath and Bardhan, 2020). Existing evidence shows that during the first week of lockdown, all India electricity consumption dropped by 22% (∼2600 GWh) as compared to the peak demand of the previous week (∼3600 GWh) (POSOCO, 2021; Prayas Energy Group, 2020). As a result, the daily electricity consumption was 20–40% lower than its corresponding value in 2019 (Aruga et al., 2020). It began to reverse from May 2020 as the unlocking began in India. However, this reversal was asymmetrical across the commercial and residential sectors (Amritha Pillay, 2020). More specifically, it was reported that an increase in summer temperatures due to heatwaves and people spending more time at home during lockdown resulted in 26% more residential electricity consumption in western India (Bielecki et al., 2021; Prayas Energy Group, 2020; PTI, 2020; Thomson Reuters, 2020). Concurrently, the ownership of air conditioners went up in the range of 22–114% compared to the pre-lockdown levels in the same region (Prayas Energy Group, 2020).

Recent studies have shown that COVID-19 is shifting the burden of energy costs to households through increased WFH and teleworking (CJ Meinrenken et al., 2020; Hook et al., 2020). Moreover, home-based working patterns influence residential energy demand by increasing energy consumption for heating, cooling, lighting, cooking, IoT devices, and other uses (Hook et al., 2020). Therefore, it is essential to accurately estimate the net energy impacts of teleworking for a country like India, where the spatial energy inequality is high within the urban areas (Gupta et al., 2020).

This paper employs a novel data-driven approach to investigate the effects of complete lockdown using NILM data from the Government of India Bureau of Energy Efficiency's residential energy use monitoring portal called the National Energy End-use Monitoring (NEEM) dashboard (BEE, 2021). We use the ‘dwelling typology’ of the residential units as an endogenous factor for exploring energy demand shifts due to WFH and heatwaves. In this purview, we hypothesise that the lockdown effects will be felt distinctly when compared with inter-dwelling and intra-dwelling effects regarding pre-pandemic electricity consumption levels. We further discuss the assumptions in detail in the methodology section. The specific research objectives of this study are, i) to investigate the shift in electricity demand as per the dwelling typologies due to nationwide lockdown in 2020 and compare it with the pre-pandemic levels (2018 and 2019); ii) to investigate the inter-effect of dwelling typologies on electricity demand shifts in the pre-lockdown and deep-lockdown stages; iii) to investigate the intra-effect of dwelling typologies on electricity demand shifts in the pre-lockdown and deep-lockdown stages; and iv) to derive data-driven policy implications on dwelling typology and demand shocks for Indian residential sector.

This study is divided into the following sections. Section 2 provides a background on the methodological approaches to NILM data analysis and its policy applications. It also expands on the energy and COVID-19 related research from the available evidence base. Section 3 explains the adopted approach in detail, focusing on machine learning-based approach using Gaussian Mixture Models. Section 4 illustrates the results and expands them into a discussion in section 5. Finally, the conclusion and policy implications for post-pandemic consumption scenarios are drawn in Section 6.

## 2 Background

### 2.1 Current NILM-based approaches for building energy management and policy design

Non-intrusive Load Monitoring (NILM) techniques are becoming a common approach for disaggregated energy consumption data acquisition. They provide a method to separate the individual consumption for certain appliances (Tabatabaei et al., 2017; Zeifman and Roth, 2011). It provides both consumer privacy and ease of implementation through already-deployed smart meters. The global push towards digitalisation has been a critical factor for the rise of NILM techniques. Digital energy programs associated with the Internet of Things (IoT), Smart Grids (SG) or Demand Response (DR) are heavily dependent on NILM methodologies for extracting information on digitised services to the end-user (Antonio Ruano et al., 2019; Hosseini et al., 2017).

A significant application of NILM techniques in residential energy management is energy efficiency decisions through itemised energy information. Such digital itemisation gives feedback to the occupant and creates ‘energy awareness’ (Hosseini et al., 2017). The non-intrusive nature of the NILM approach and its easy utilisation through smart meters are its major advantages over conventional energy metering methods. NILM has also emerged as a critical digital technology for healthcare management in the pandemic for vulnerable population groups. In a residential energy management context, NILM has two major applications in Home Energy Management Systems (HEMS) and Ambient Assisted Living (AAL) (Antonio Ruano et al., 2019). Detailed literature review of NILM techniques can be referred from the following papers (Antonio Ruano et al., 2019; Hosseini et al., 2017; Tabatabaei et al., 2017; Zeifman and Roth, 2011).

The main stages are NILM data analysis, as reported by Antonio Ruano et al. (2019), categorised into four distinct stages. It involves data collection, event detection, feature extraction and load identification. Our study relies entirely on the NEEM dashboard (BEE, 2021) for data collection and conducts event detection according to the dwelling typologies. Existing NILM literature classifies an event as any switch in a signal from a certain steady-state to a new state. Event detection typically consists of expert heuristic, probabilistic models and matched filtering (Anderson et al., 2012). Expert heuristics were a common approach in the 1990s and 2000s that involved creating a set of rules for each appliance (Hosseini et al., 2017). State-of-the-art methods in NILM event detection are probabilistic models that can be evaluated through supervised or unsupervised approaches (Liu et al., 2019). For example, Decision Trees (DT) and Long Short-Term Memory (LSTM) models are used for event detection with over 98.6& and 92.6% accuracy, respectively (Le and Kim, 2018). Unsupervised classification and clustering algorithms are also used as a novel approach to event detection that pushes the boundaries of AI in NILM-based energy demand analysis (Alcalá et al., 2017; Beckel et al., 2014; Bonfigli et al., 2015).

Unsupervised approaches define the current practical applications of the NILM approach for home energy management cases (Antonio Ruano et al., 2019; Hosseini et al., 2017). Over the last ten years, research efforts have been focused on the development of real-time disaggregation algorithms for inferring the state of individual appliances and indicating the total energy consumption in an unsupervised manner (Abubakar et al., 2017; Antonio Ruano et al., 2019; Zoha et al., 2012). Load classification and load separation are two main types of unsupervised categorisation approaches in NILM literature. Recent systematic literature review of the unsupervised NILM classification can be referred here: (Abubakar et al., 2017; Bonfigli et al., 2015; Liu et al., 2019). Methodologically, state-of-the-art NILM algorithms have been proposed using variants of Hidden Markov Models (Jia et al., 2015; Kim et al., 2011; Kolter and Jaakkola, 2012; Makonin et al., 2016), Graph Signal Processing (Sandryhaila and Moura, 2014; Zhao et al., 2015) and deep learning (Kelly & Knottenbelt, 2015, 2015, 2015; Zhang et al., 2019).

In cases where the power level of each appliance is not known, researchers have used event-based blind disaggregation algorithms using Gaussian mixture models (GMM) for clustering to automatically detect appliances from the aggregate data (Qureshi et al., 2021). The benefit of using GMM over other clustering methods is that they can automatically learn the statistical distributions present in the data (Abubakar et al., 2017; Antonio Ruano et al., 2019). However, present evidence shows that the existing disaggregation techniques often need some supervised learning and parameter tuning (Hosseini et al., 2019; Ridi et al., 2016). Therefore, for the first time, Qureshi et al. (2021), proposed a fully blind event-based disaggregation method using GMM. Furthermore, we innovatively build on their approach to disaggregate building energy use based on dwelling typology to explore the combined effect of lockdowns and heatwaves at a national scale for Indian cities.

Very limited evidence exists on the role of NILM-based approaches in energy policy design. Recently, International Energy Agency emphasised on the NILM as a digitalisation solution for better energy efficiency at a power system level. The authors emphasised on leveraging NILM at the policy impact monitoring and policy design stages (Jeremy Sung et al., 2021). Policy experiments were funded by the UK Government under the Smart Meter Enabled Thermal Efficiency Ratings (SMETER) Innovation Programme to better understand the role of NILM in measuring residential thermal performance to inform future policy (GOV.UK, 2018). Klemenjak et al. (2020) further stated that as a policy tool NILM can be used to perform diagnostics of household appliances and industrial components that can reduce operational costs. Similarly, Salani et al. (2020) in context of Swiss Government funded Lugaggia Innovation Community (LIC) project showed that NILM can be used effectively in distributed energy planning. NILM was also discussed as a critical public health tool through its integration in Ambient Assisted Living (AAL). AAL is the use of sensor-based intelligence to support people who needs critical care. It involves NILM of daily activities, monitoring the health deterioration at long term or producing alerts for short-term interventions (Hernández et al., 2019). Nonetheless, with advancement of sensor intelligence and computational methods, the critical policy design challenge lies with the privacy concerns of such monitored datasets that needs rigorous ethical assessment (Sangyoung, 2020).

### 2.2 COVID-19 effect on residential energy demand in India

The International Energy Agency (IEA) stated that electricity demand dropped quickly across Europe and India under lockdown but steadily recovered as measures were gradually softened. By July 2020, the weather corrected electricity demands stayed 5% below the 2019 levels for the same month in most countries, except in India, where the recovery was more pronounced (IEA, 2021a). Data from India's National Load Dispatch Centre shows that all India electricity consumption dropped by 22% in the first week of lockdown (25 March – April 1, 2020) compared to the previous week's peak (POSOCO, 2021; Prayas Energy Group, 2020). In contrast, during the lockdown, residential electricity consumption is expected to increase as people spend more time at home and summer temperatures. Although, the actual impact on electricity consumption is not yet clear (Prayas Energy Group, 2020).

A recent survey showed that in 81 households in the Indian states of Maharashtra and Uttar Pradesh, the average daily increase in electricity consumption was 26% more than the pre-lockdown levels (Prayas Energy Group, 2020). In addition, it showed that households with air conditioning (AC) showed an average daily increase in the range of 45–60%. On the other hand, the households without AC showed a 35–114% increase in average electricity consumption (Prayas Energy Group, 2020). This study also reported a 2–3 °C higher maximum temperature in the survey areas compared to the 2019 levels (Prayas Energy Group, 2020).

A similar study involving single-phase smart meters in 93 urban households in Uttar Pradesh showed that electricity use was lower during the first three weeks of May 2020 (i.e., during the deep-lockdown period) but exceeded past consumption levels after 23 May (Pathak et al., 2020). The study reported fewer power cuts during May 2020 (43.5 min/day/HH) than in May 2019 (72.5 min/day/HH). It was also reported that the relative drop in electricity use was highest amongst households with AC. Occupants’ interviews revealed that lower use of AC was due to adherence to public advisories against the use of AC and desert coolers to prevent the spread of coronavirus and also due to inability to get AC repaired during lockdown (Pathak et al., 2020; Shalu Agarwal et al., 2020). Occupants also revealed limiting usage of AC to lower the electricity bills during the lockdown as it was causing significant financial stress (Pathak et al., 2020; Shalu Agarwal et al., 2020).

Aruga et al. (2020) found that the poorest states in India did not recover well compared to the wealthier states when nationwide lockdown began to relax. The authors used an autoregressive distributive lag modelling approach to show that regions with higher income levels were more likely to recover their energy consumption to pre-lockdown levels faster than those with lower income levels (Aruga et al., 2020). However, their analysis did not disaggregate residential and commercial consumption, which remains its limitation. Our study presents such disaggregated analysis at an urban residential scale that can inform post-pandemic energy efficiency policies and strengthen India's recovery efforts. This study also informs how demand-side digitalisation using tools like smart meters can improve energy policy decision-making especially in low-and-middle income countries.

## 3 Data and methods

**Fig. 1:**
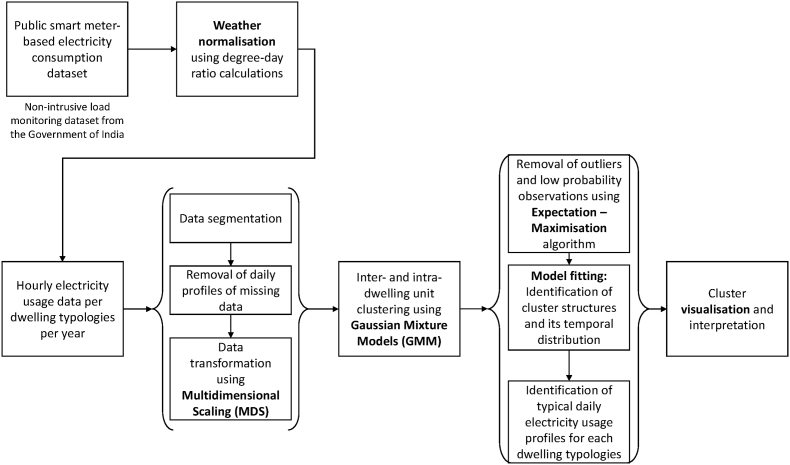
Methodological framework of this paper.

The overall data-driven methodological approach is illustrated in Fig. 1. It consisted of three key steps: i) Public non-intrusive load monitoring data collection and its pre-processing for weather normalisation, ii) Extraction of hourly electricity consumption data as per the dwelling typologies, its segmentation and dimensionality reduction and iii) Identification of typical daily electricity usage profiles as per the inter- and intra-dwelling units using Gaussian Mixture Modelling.

### 3.1 Data source

Non-Intrusive Load Monitoring (NILM) data was collected through the Bureau of Energy Efficiency (BEE), Government of India's online public portal called National Energy End-use Monitoring (NEEM) dashboard (BEE, 2021). BEE reports NILM smart meter data of electricity end-use of 200 households across 13 cities in India at an hourly resolution (see Fig. 1). Detailed sample specifications are presented in Table A1 in Appendix. We collected 153 days data between March and July for pre-COVID (2018 and 2019) and deep-COVID (2020) periods. The specific timeframe of March to July accommodates this paper's scope of evaluating nationwide lockdown impacts on residential electricity consumption.

The dataset consisted of the 24-h load profile of the urban households based on their dwelling type. The data was pre-segmented into five dwelling typologies, namely, 1-bedroom (BR1), 2-bedroom (BR2), 3-bedroom (BR3), more than 3-bedroom (M3BR) and 1-room units (RM1) (BEE, 2021). We follow this nomenclature consistently throughout this study. The final dwelling-typology based sampling specifications are presented in Table A2 in the Appendix.

BEE reports specific attributes to the NILM datasets on the NEEM website (BEE, 2021). For example, it states that higher weightage was given to upper and middle-income consumer profiles, and inverse allocations were given to lower-income bands. Therefore, the scope of monitoring was directed towards upper and middle-income socioeconomic classes (SECs) (BEE, 2021). To overcome this data scoping limitation, we based our analysis on dwelling typologies as a key non-income driver (Debnath et al., 2019) of electricity demand in urban India. In addition, contrary to the common seconds or hertz (Hz) level granularity of the NILM dataset, BEE provides the data at hourly resolution at a whole building/dwelling-unit energy use level (BEE, 2021).

**Fig. 2:**
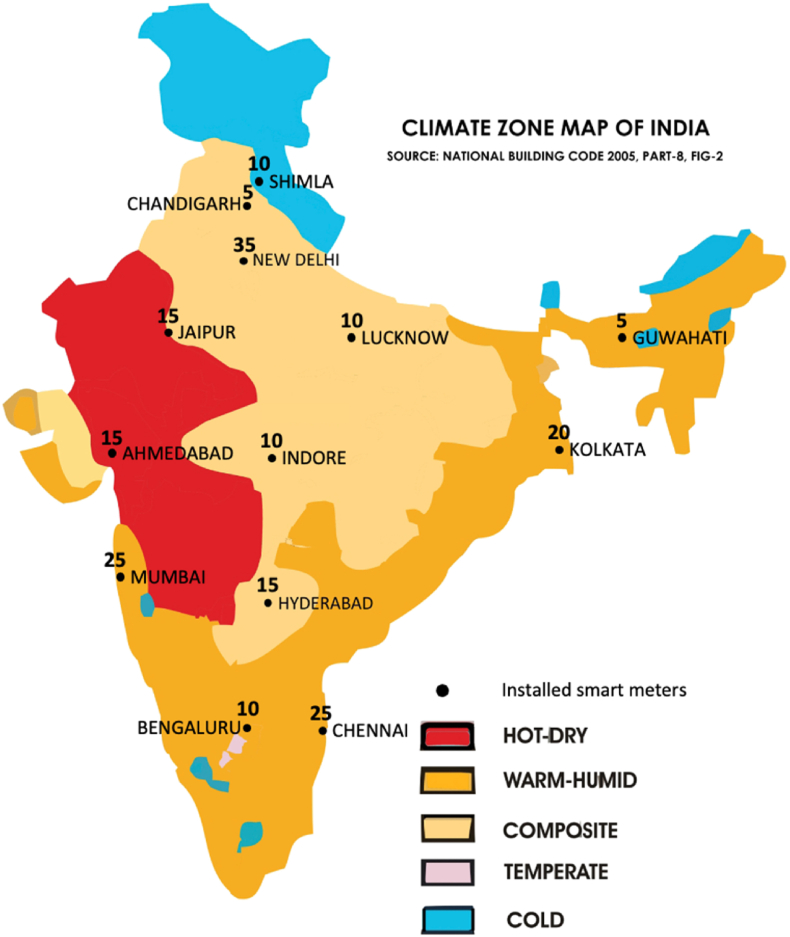
Non-intrusive load monitoring locations across 13 cities and five climatic zones in India (Source: BEE, 2021).

Total data points representing electricity demand (in kilowatt-hour (kWh) between March–July for the target years across the dwelling typologies were 52,107. We adopted the ratio based Degree-Day Normalisation method for weather normalisation of the NILM datasets (ASHRAE, 1985). It generated weather normalised electricity consumption by factoring out the effect of outdoor temperature. Thus, enabling comparison for electricity demand across five climate zones (see Fig. 2). The normalisation isolated the effects of weather change on energy performance by using the heating degree-day (HDD) and cooling degree-day (CDD) values.

Degree-days represent the absolute value of the difference between a reference or base temperature of a given time (Beheshti et al., 2019). Bhatnagar et al. (2018) estimated the reference temperature for India, i.e., base temperature for cooling is 18.3 °C and heating is 17.4 °C, with an average base temperature for cooling and heating 18 °C. The normalised electricity consumption was calculated using eq (1) with 18 °C as the base reference temperature.

**Fig. 3:**
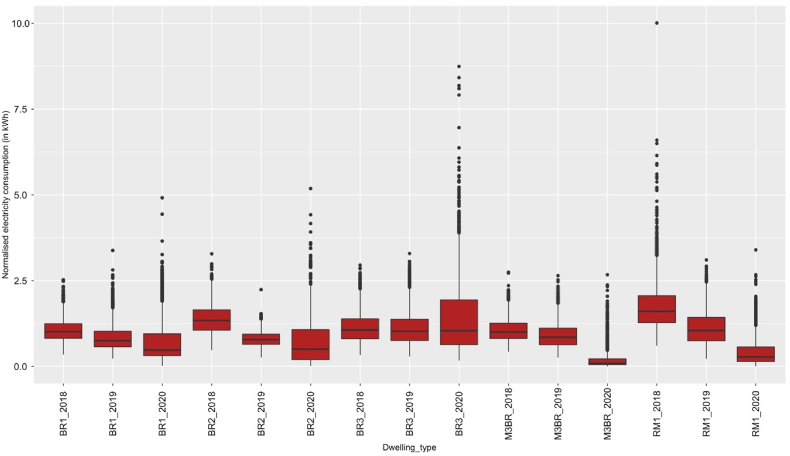
Spread of the weather normalised electricity demand in kilowatt hour (kWh) across dwelling type for the analysis period (Mar–July) in 2018, 2019 and 2020.

Eq. 1 $$E_{normalised}=\frac{E_{actual}}{{\left(HDD+CDD\right)}_{actual}}\times {\left(HDD+CDD\right)}_{average-year}$$

where $E_{normalised}$ = Normalised energy consumption; $E_{actual}$ = Actual energy consumption; ${\left(HDD+CDD\right)}_{actual}$ = Actual hourly heating degree day/cooling degree day of the energy use; ${\left(HDD+CDD\right)}_{average-year}$ = “average year” degree-day value over 3 years (2018,2019, and 2020) for each day. The weather normalised electricity consumption data (in kWh) is illustrated in Fig. 3.

[Note: x-axis shows dwelling type in each year. BR1_2018 = 1-bedroom unit in 2018; BR2_2018 = 2-bedroom unit in 2018; BR3_2018 = 3-bedroom unit in 2018; M3BR_2018 = More than 3-bedroom unit in 2018, and RM1_2018 = 1-room unit in 2018. This nomenclature is followed for 2019 and 2020 as well].

### 3.2 Multi-dimensional scaling (MDS)

The NILM data presented above have high dimensionality that was computationally costly for training the Gaussian Mixture Models (GMM). Here, we have used a multi-dimensional scaling (MDS) technique to reduce the dimensionality of the input data before performing the clustering analysis for reducing the computational costs. A similar approach was also suggested by (Bouveyron and Brunet-Saumard, 2014; K. Li et al., 2018). MDS is a robust dimensionality reduction technique that retains vital information in the raw data as compared to other well-known dimensionality reduction techniques such as piecewise aggregate approximation (PAA) and piecewise linear approximation (PLA) (T.F. Cox & M.A. Cox and Cox, 2020). In addition, MDS retains more useful information about the pairwise distance among the data points, which is critical for GMM-based clustering (R. Li et al., 2016). Thus, it is a widely used method for data pre-processing and visualisation of cluster analysis.

To apply MDS to a *q*-dimensional raw dataset, each observation in the raw dataset was considered a point in the *q*-dimensional space. First, the distance matrix *M* was calculated for all pairwise distances among the points. Then, all the points in the original *q*-dimensional were projected into a *p*-dimensional space (*p < q*), such that the distance matrix of the points in the *q*-dimensional space *M’* is similar to *M* as much as possible. Thus, the dimensionally reduced data can be reached by considering each point in the *p*-dimensional space as an observation in the *p*-dimensional dataset.

The dissimilarity between *M* and *M’* is measured using stress as defined in eq (2) (T.F. Cox & M.A. Cox and Cox, 2020), while the detailed working procedure was adopted from (W.S. Torgerson, 1952),

(2) $$Stress=\;\sqrt{\frac{\begin{array}{l} \sum_{i=1,\;j=1}^{N}{\left(d_{i,j}^{\prime }-d_{i,j}\right)}^{2}\;\\ i\neq \;j \end{array}}{\begin{array}{l} \sum_{i=1,\;j=1}^{N}d_{i,j}^{2}\;\\ i\neq \;j \end{array}}}$$

where $d_{i,j}$ and $d_{i,j}^{\prime }$ denote the distances between the *i*th and *j*th points in *M* and *M′*, respectively.

### 3.3 Model-based clustering using Gaussian Mixture Models (GMM)

Model-based clustering belongs to the category of unsupervised machine learning algorithms. It is soft partitioning where observations could exist in several clusters rather than be assigned strictly to a single cluster. Soft partitioning or clustering presents an advantage over hard clustering methods like K-means by estimating uncertainty measures about how much a data point is associated with a specific cluster (Kevin P. Murphy, 2012). The core assumption with this clustering approach is that there are *k* mixture components (i.e., clusters) in some feature space that comprise a mixture of probability distributions, *p(x)* (Waggoner, 2020).

Gaussian mixture models (GMM) are a special class of finite mixture models, where each component, *k,* is assumed to be normally distributed. In deciding to fit a GMM for clustering, we are concerned with classifying electricity demand across various dwelling typologies (*observations*) into components (i.e., clusters). Therefore, we answer the following question from an unsupervised machine learning framework: *what, if any, grouping exits in the dwelling type-based electricity demand feature space?*

Formally, the probability distribution, *p(x)*, is comprised of the sum of all normally distributed components, k, see eq. (3) (after (Figueiredo and Jain, 2002)),

(3) $$p\left(x\right)=\overset{G}{\underset{k=1}{\sum }}\alpha_{k}N\left(x;\mu_{k}\sigma_{k}\right)$$

where, $\alpha_{k}$ is the probability weight, or mixture size, for component *k,* driving the assignment of observations to components, where $\overset{G}{\underset{k=1}{\sum }}\alpha_{k}=1$. The joint probability distribution, *p(x)*, is defined by the weighted average of all individual components, *k*. The, $N\left(x;\mu_{k}\sigma_{k}\right)$ represents that in GMM we assumed a Gaussian distribution with $\mu_{k}$ representing mean and $\sigma_{k}$ showing the variance.

**Fig. A1:**
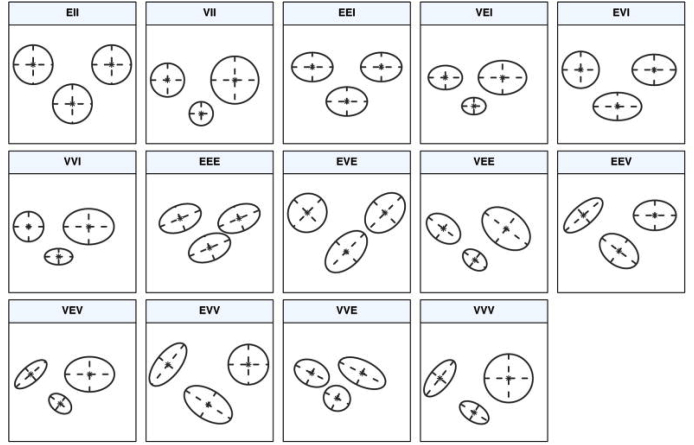
Ellipses of isodensity for each of the 14 Gaussian models obtained by eigen-decomposition in case of three groups in two dimensions (Source: (Scrucca et al., 2016)).

The soft clustering assignment of the observations based on the normal probabilistic distribution of GMM allows overlapping between components. The similarity between observations assigned to a component is now defined by similarities in the probability of observations being assigned to a given cluster, *k*. The mean ($\mu_{k}$) and variance ($\sigma_{k}$) describe the shape of the components, and the complexity of the feature space, *p(x)*. In a multivariate setting, there are 14 possible Gaussian models with different geometric characteristics through the parametrisation of volume, orientation, and shape of ${\text{Σ}}_{k}$. We use mclust version 5.4.3 (Scrucca et al., 2016) in R version 3.5.3 for model estimation. The characteristic models are illustrated in details in Table A3 and Fig A1 in the Appendix (Scrucca et al., 2016).

The GMM fit criteria are to estimate the values of $\alpha_{k}$, $\mu_{k}$ and $\sigma_{k}$ to ensure that the GMM has the maximum-likelihood. The Expectation – Maximisation (EM) algorithm is commonly used to fit GMM. It produces ML estimates of parameters when there is a many-to-one mapping from an underlying distribution to the distribution governing the observation.

The implementation of EM consisted of three steps – initialisation, expectation step and maximisation step (as per (Figueiredo and Jain, 2002; Moon, 1996)). The initialisation step consisted of a random selection of parameters based on the number of components set at the initialisation step. An iterative step of expectation (E) and maximisation (M) step was then conducted to improve the estimation of model parameters.

Each observation was assigned to one of the mixture components in the E step to assign the highest probability to this observation (Moon, 1996). Then the relative probability of each observation, *i*, belonging to all possible components, *k*, is calculated and ranges between [0,1]. High values indicate the *k*th component is a good fit for *i*, and low values suggest the *k*th component is a poor fit for *i*. Based on this probability feature, the parameter ($\alpha_{k}$, $\mu_{k}$ and $\sigma_{k}$) of each mixture component, *k*, was updated in the M step. The algorithm was assumed to have converged when the updated parameters of all mixture components do not change further, and the EM step was terminated.

The robustness of the clustering results was further improved by using the modified EM, proposed by (Banfield and Raftery, 1993; Muthén and Shedden, 1999). The modified algorithm automatically identified and removed the low probability observations in the mixture components. More recently, a similar approach was adopted by (K. Li et al., 2018).

The optimal number of mixture components, *G*, was determined using the Bayesian Information Criterion (BIC) (Hsu, 2015; Neath and Cavanaugh, 2012). BIC is one of the most widely used tools for statistical model selection in GMMs. The lowest value of BIC is preferred for the GMM with optimal *G* value. In case of negative BIC numbers, the value that has the largest modulus indicated the preferred model. Thus, for both inter-and intra-dwelling clustering, the *G* value, which can minimise BIC, was used as the optimal *G* number of GMM fitting. Existing evidence shows that optimal clusters for single inter-building typical energy usage generally ranges from 2 to 8 (Ma et al., 2018; Rhodes et al., 2014; Yang et al., 2017), while for intra-building clustering, it ranges from 2 to 14 (K. Li et al., 2018). Relevant codes can be accessed from https://github.com/Ramit1201/EnergyProp.

## 4 Results

### 4.1 Shifting of daily residential load-curves

**Fig. 4:**
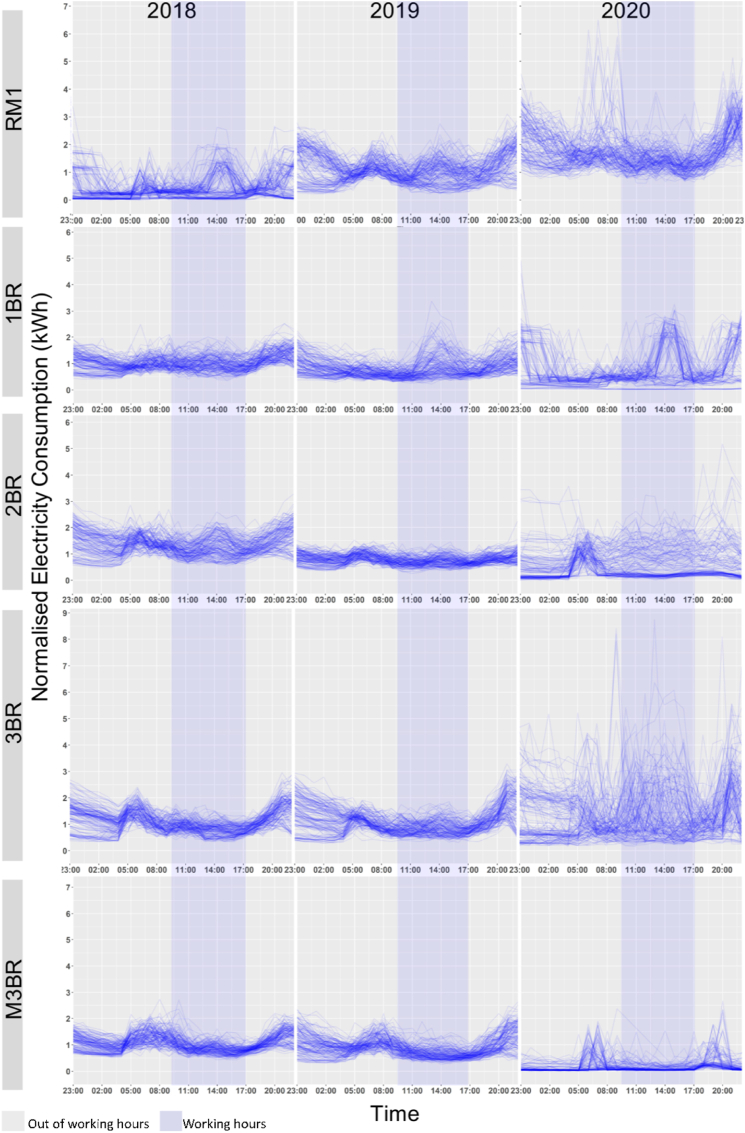
Daily load curves of dwelling typologies no lockdown (2018–2019) and deep-lockdown (2020) periods.

The load profiles for the dwelling typologies can during their typical office working hours (09:00–17:00) and out of working hours is illustrated in Fig. 4 for 2020, 2019 and 2018. It shows significant shifts in the residential load curves for 2020 as compared to 2018 and 2019. For 2020, the peak demand appears to occur stochastically during the daytime across the dwelling units, which is missing for the 2018 and 2019 curves (see Fig. 4).

The RM1 is 2020 observed its maximum peak demand (∼6.56 kWh) between 05:00–11:00 h. A similar trend follows between 20:00–23:00 h. In the same period, the maximum demand for 2019 was approximately 2.5 kWh and 2 kWh for 2018, which peaked at ∼2.95 kWh for both the years during a typical office work hour (09:00–17:00) (see Fig. 4). Thus, the work-from-home (WFH) impact for one-room dwelling units (RM1) can be evaluated by a rise in maximum peak demand by approximately 125% during daytime and approximately 100% during night-time.

**Fig. 5:**
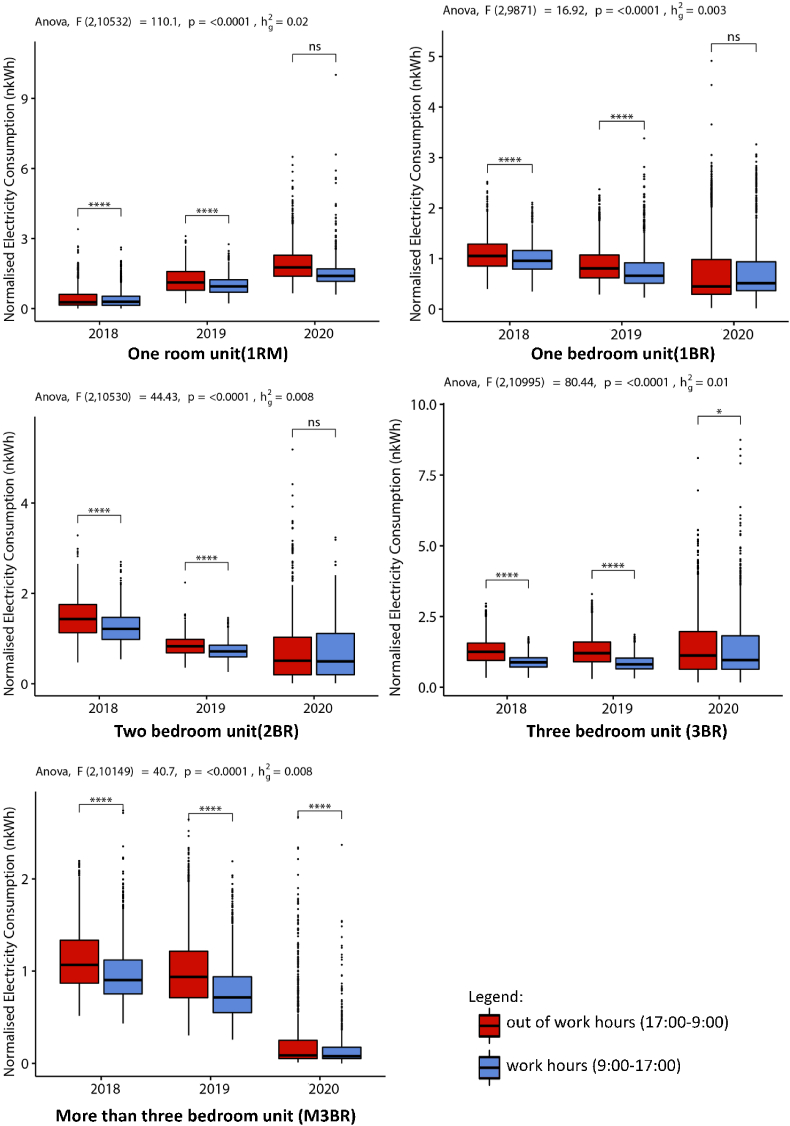
Variance in daily electricity demand for work and out-of-work hours during deep-lockdown (2020) and non-lockdown period in 2018 and 2019 [Note: ns indicates p > 0.05; * indicates p ≤ 0.05; ** indicates p ≤ 0.01; *** indicates p ≤ 0.001].

In addition, Fig. 5 further shows that for 2020 RM1 electricity demand, there was no significant variance between working and non-working hours, whereas significant variance existed for 2018 and 2019 electricity demand profiles. Thus, it further illustrates that work-from-home norms in RM1 is shifting the working hour-led electricity consumption patterns, and thus, affecting overall load curves.

Interestingly for the one-bedroom unit (1BR) case, while the peak demand during daytime for 2019 and 2020 remain identical (∼3.48 kWh), the peak appears more frequently between the working hours for 2020 (see Fig. 4). However, the load curves for 2020 also show an emergence of maximum night-time peak (∼5 kWh) between 20:00–23:00, which is a 43.67% rise for the same period in 2018 and 2019. Like the RM1, we did not find any significant variance (see Fig. 5) between the demand for working and out of working hours for BR1, indicating the impact of WFH norms on daily practices.

A substantial distortion of the load curves for two-bedroom units (2BR) in 2020 was also observed in Fig. 3. During non-working hours the maximum peak demand was ∼5.38 kWh in 2020, compared to ∼2 kWh for 2019 and 2018 (see Fig. 4). However, the variance in the working and out of work hours was not significant for 2020, as mentioned above (see Fig. 5). Thus, demonstrating changing electricity demand patterns in 2BR typologies due to WFH.

The peak shifts in three-bedroom units (3BR) were even more stochastic throughout the day for the lockdown period in 2020, as shown in Fig. 4. The maximum peak demand for work hours was ∼8.98 kWh in 2020, a ∼290% increase in peak demand from 2019 to 2018 levels (∼2.30 kWh). For the out-of-work hours, the maximum demand peaked at ∼8.10 kWh in 2020, while ∼2.58 kWh in 2019 and ∼2.32 kWh in 2018. In contrast to the other dwelling typologies, the variances in the working and non-working hour peak demands for 2020 in BR3 is significant at 95% CI (see Fig. 5). Similarly, for more than three-bedroom units (M3BR), the variance in the peak electricity demand for work and out-of-work hours in 2020 is significant at 99.9% CI (see Fig. 5). In addition, the load curves and the peak demand was identical for 2020, 2019 and 2018 (see Fig. 4).

The 3BR and M3BR dwelling units represent a typical urban residential typology of upper-middle and higher-income Indian households. Significant variances in the peak demand shifts during work and out-of-work hours in lockdown show that work-from-home norms did not substantially affect the load curves in 2020 compared to 2019 and 2018 (see Fig. 5). The median electricity consumption generally showed a decreasing trend over the lockdown (2020) and non-lockdown period (2019 and 2018), as illustrated in Fig. 5.

**Fig. 6:**
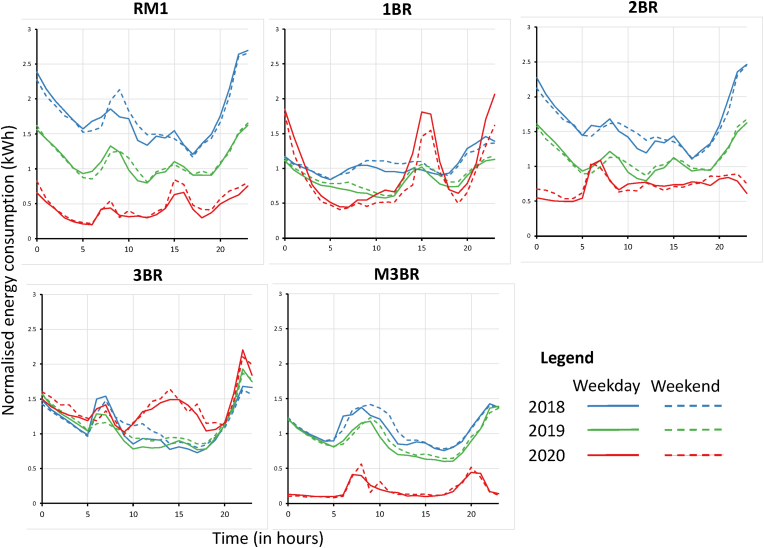
Weekday versus weekend aggregated energy demand (in kW) across the residential typologies. The y-axis represents the hour of the day (24-h scale).

Furthermore, a cross-sectional view of residential energy demand during the weekend and weekdays shows a decrease in consumption for RM1 and M3BR for 2020 compared to 2018 and 2019 (see Fig. 6). However, there are distinct shifts in hourly load profiles for all the housing typologies in 2020. For example, peak demand for RM1 happens at 16:00 h for the weekdays in 2020 while 15:00 h for the weekend (see Fig. 6). As a result, the peak demand for 2020 weekdays is ∼55.45% lower and ∼27.30% lower for the weekend compared to the 2018 and 2019 levels. For 2BR typologies in 2020, Fig. 6 shows a peak shift and decrease in demand, i.e., ∼45.50% for weekdays and ∼9.09% for the weekend (compared to 2018 and 2019 values). Similar, decreasing peak shifts were observed for M3BR units. As a result, the weekend consumption for M3BR is ∼163% and ∼222% (weekday) lower than that of the 2018 and 2019 levels (see Fig. 6).

In contrast, a rise and shift in peak demand are observed for 1BR units in 2020, which is ∼85% increase for weekdays and ∼55% increase for weekend consumption levels compared to the 2018 and 2019 values (see Fig. 6). A similar pattern is observed for 3BR, with a distinct shift in the daily load curve between 09:00–16:00 (see Fig. 6). The rise in weekend peak demand for 3BR is around 6.77%, where the demand change is negligible for weekdays compared to the 2018 and 2019 values (see Fig. 6). These cross-sectional results further demonstrated that different housing typologies experienced lockdown related demand shifts across India. Section 4.2 presents the granular results of these shifts using the GMM-clustering approach (as described in section 3.3).

### 4.2 Intra- and inter-dwelling unit clustering

**Fig. 7:**
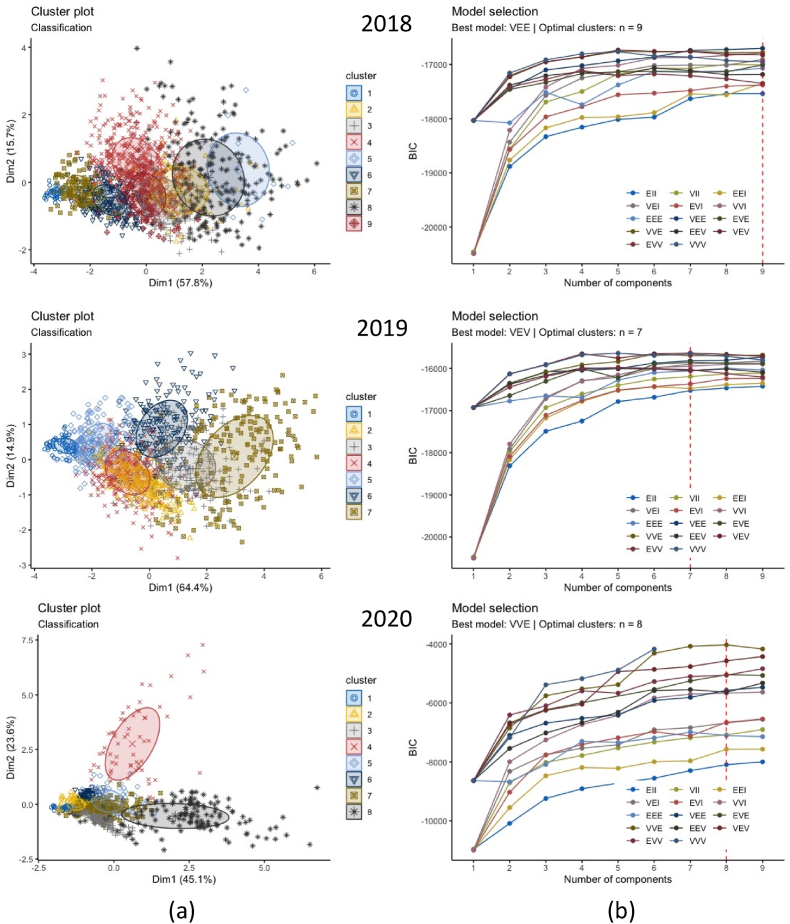
Derived cluster profiles at an inter-dwelling electricity demand. (a) Elliptical shape and the coloured boundaries denote the Gaussian clusters with outliers derived in two-dimension; (b) Bayesian Information Criteria (BIC) plots for 14 models fitted to the electricity consumption data showing the geometric characteristics of this multidimensional data and its covariance parametrisation. [Note: In one dimension, there are just two models: E for equal variance and V for varying variance. In the multivariate setting, the volume, shape, and orientation of the covariances can be constrained to be equal or variable across groups that result in 14 models (see Appendix: Fig A1 and Table A3).].

Table 1 shows the GMM model fit summary with Bayesian Information Criterion (BIC) and log-likelihood values and the determinant model for clustering the electricity consumption of the inter-and intra-dwelling types. The clustering profiles were extracted for the March–July in pre-pandemic (2018 and 2019) and nationwide lockdown stages (2020), as mentioned in section 1. The inter-dwelling type models illustrate 5-dimensional features based on the year and the dwelling units. In contrast, the values for intra-dwelling types show a single-dimensional model as it represents each dwelling typology in a specific year. Most of the clusters identified were elongated ellipses with outliers (see Fig. 7a) that supports our methodological assumption that hard-clustering approaches like *k*-means may not have worked well for our dataset. *K*-means has no built-in way of accounting for oblong or elliptical clusters (P.-N. Tan et al., 2005).

**Table 1:** GMM model fit summary with BIC values for inter- and intra-dwelling type.

Sl. no	Inter-dwelling type					
	Model name	Best model	Optimal cluster (G)	Bayesian Information Criteria (BIC)	Log likelihood	Sample (n) [NA values are omitted]
1	2018	VEE	9	−16701.19	−8074.27	1439
2	2019	VEV	7	−15634.19	−7373.47	1440
3	2020	VVE	8	−4031.27	−1693.30	770
	**Intra-dwelling type**					
4	BR1_2018	V	3	−1390.21	−668.52	770
5	BR1_2019	V	2	−1081.23	−511.52	1440
6	BR1_2020	V	4	−905.87	−2151.75	770
7	BR2_2018	V	2	−1630.86	−775.43	1439
8	BR2_2019	E	5	−189.53	−116.58	1440
9	BR2_2020	V	6	−612.41	−269.65	770
10	BR3_2018	V	3	−1558.88	−761.26	1439
11	BR3_2019	V	2	−2002.59	−927.20	1440
12	BR3_2020	V	2	−1381.77	−654.30	770
13	M3BR_2018	V	2	−713.10	−338.37	1439
14	M3BR_2019	V	3	−1053.66	−497.74	1440
15	M3BR_2020	V	6	−1925.94	1039.40	770
16	RM1_2018	V	3	−2582.27	−3468.70	1439
17	RM1_2019	E	2	−2053.39	−995.81	1440
18	RM1_2020	V	6	40.91	47.04	770

[Note: Optimal cluster (G) denote the value that fitted the GMM model value with lowest BIC values. Optimal G-values is in the range of 2–8 for inter-building clustering and a range of 2–14 for intra-building clustering. Log-likelihood values is used to validate the BIC-driven model fit. It is a function of sample size (n), and a higher value determines better fit (see section 3.3 for detail)].

VEE was estimated to be the best model for the 2018 data, indicating nine-component mixtures with covariances having varying shape volume and orientation (see Fig. 7b). For 2019, VEV was estimated to be the best model fit with seven-component mixtures with covariances having variable volume and orientation but the equal shape (see Fig. 7b). Similarly, for 2020, the best BIC model fit was obtained for VVE. It shows eight-component Gaussian mixtures with covariance having variable volume and shape and equal orientation (see Fig. 7b). It is to be noted that these model fits are obtained at an inter-dwelling unit scale. Their varying shape characteristics denote that there were some differences in the residential electricity demand for each year.

**Fig. 8:**
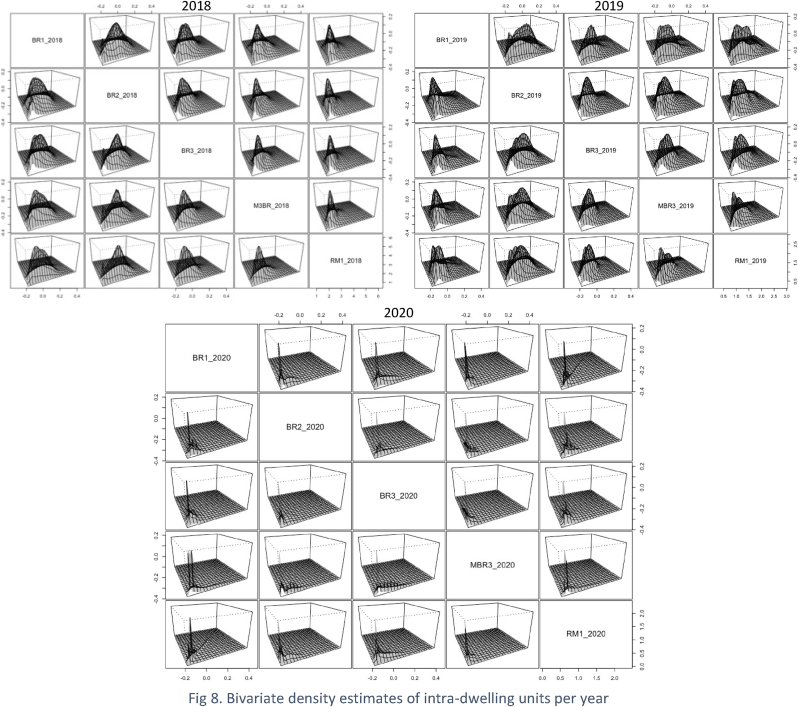
Bivariate density estimates of intra-dwelling units per year.

Table 1 further illustrated that the number of data points (n) for these years were different, which may have also contributed to the mixture components’ shape, volume, and orientation characteristics. We further investigate the impact of lockdowns at an intra-dwelling scale that shows the granularity of demand shifts across the dwelling typologies for 2018, 2019 and 2020. Fig. 8 illustrate the bivariate density estimates for the intra-dwelling units. The sharp peaks in 2020 represent the concentration of data points for all the dwelling typologies that further supports our hypothesis that lockdowns significantly impacted the residential electricity demand through work-from-home.

**Fig. 9:**
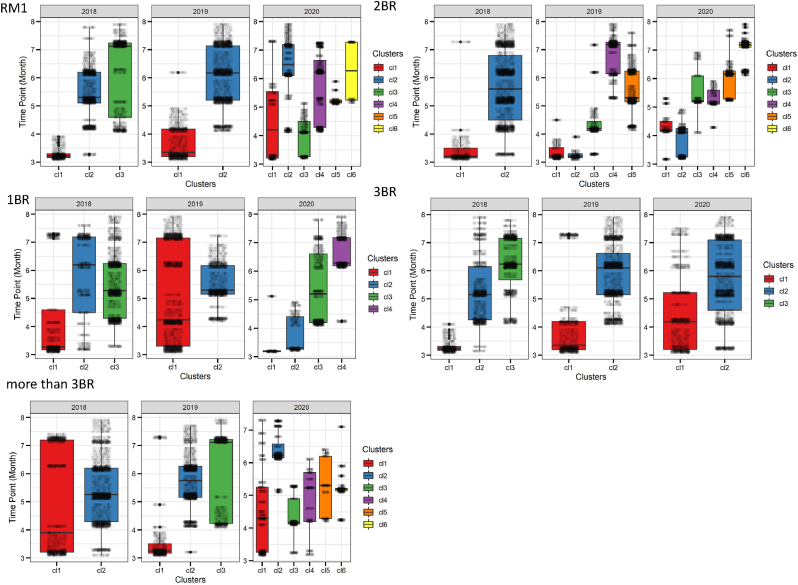
Temporal distribution of cluster structures demonstrating the variance in clusters (x-axis) across 2018, 2019 and 2020. The y-axis shows month from March (3) to August (8) as time points.

Fig. 9 shows the time point distribution of the cluster structures across the residential typologies across month. Consumption patterns for RM1, 2BR and more than 3BR (M3BR) housing units had more clusters for 2020 than the other years, illustrating greater variance in energy demand behaviour during the lockdown period (March–August, see Fig. 9). It is to be noted that the NEEM database is limited to lower-middle (RM1 and 1BR) to higher-middle-income (3BR and M3BR) household categories (see section 3), this result implicated that the consumption pattern shifts were more profound in the lower-middle- and middle-income dwelling typologies. Further typology-based granular details of the results are presented below.

#### 4.2.1 One-room unit (RM1)

**Fig. 10:**
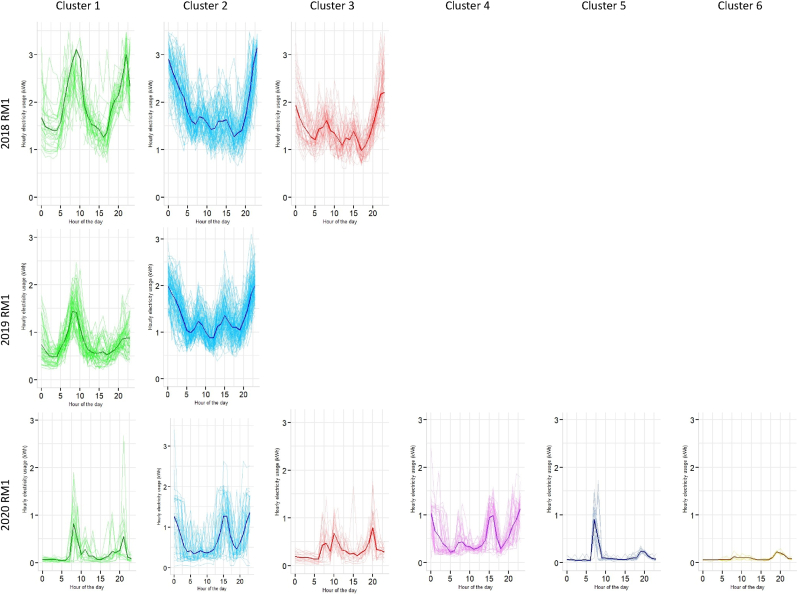
Extracted clusters of electricity demand with mean curves for one-room units (RM1) [Note: y-axis shows weather corrected energy demand in kWh].

Table 1 shows that the three mixture components were extracted for RM1 in 2018 and two clusters for 2019, while six clusters for 2020. The cluster memberships are illustrated in Fig. 10, where for 2018 and 2019, the mean electricity demand follows a similar trajectory in cluster 1 and cluster 2. Cluster 1 shows a peak around 10:00 h, while cluster 2 shows series of the trough. However, this pattern is completely altered for 2020, with five clusters showing peaks at different times of the day (see Fig. 10). In 2020, peaks were more frequent around 09:00–10:00 h, 14:00–15:00 h and 20:00–23:00 h with a mean demand around ∼1.5 kWh. This shift further illustrates the effect of work-from-home on electricity demand in one-room dwelling units in urban India (see demand across the lockdown period for 2020, as shown in Figs. 4 and 5).

#### 4.2.2 One-bedroom unit (BR1)

**Fig. 11:**
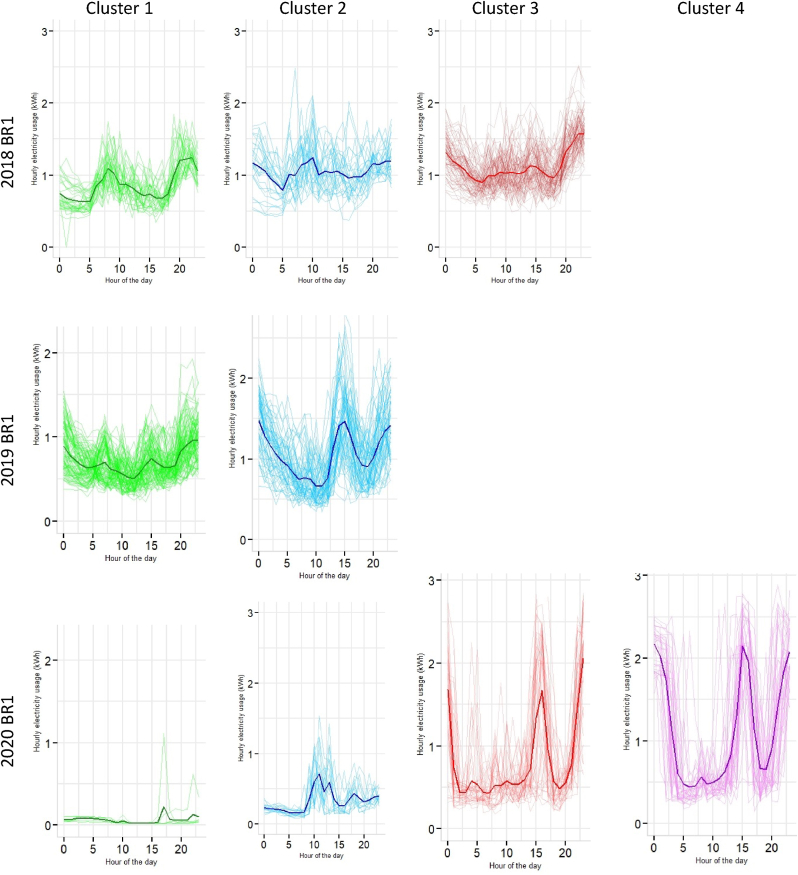
Extracted clusters of electricity demand with mean curves for one-bedroom units (BR1).

Three mixture components were extracted for 2018, two for 2019 and four for 2020 electricity demand profiles of BR1 dwelling typology (see Fig. 11 and Table 1). Energy demand peaks twice in cluster 1 for 2018 at 09:30 (∼1.78 kWh) and 22:00 h (∼1.94 kWh), where the other two clusters project troughs in the hourly load curves. No significant demand peaks were observed in cluster 1 for 2019 and 2020 (see Fig. 11). However, cluster 2 for 2020 show a rise in demand for the period between 10:00 and 17:00 h, emulating the demand for working hours (see Fig. 4). A similar trend was observed for cluster 2 in 2020 as well, between 09:00–15:00 h. In contrast, cluster 3 and cluster4 for 2020 have a massive surge in electricity demand between 12:00 and 17:00, with peaks reaching up to 2.89 kWh (see Fig. 11). It further illustrates the stochastic shift in load curves due to WFH practices, also demonstrated through the time point distribution in Fig. 9.

#### 4.2.3 Two bedroom-unit (BR2)

**Fig. 12:**
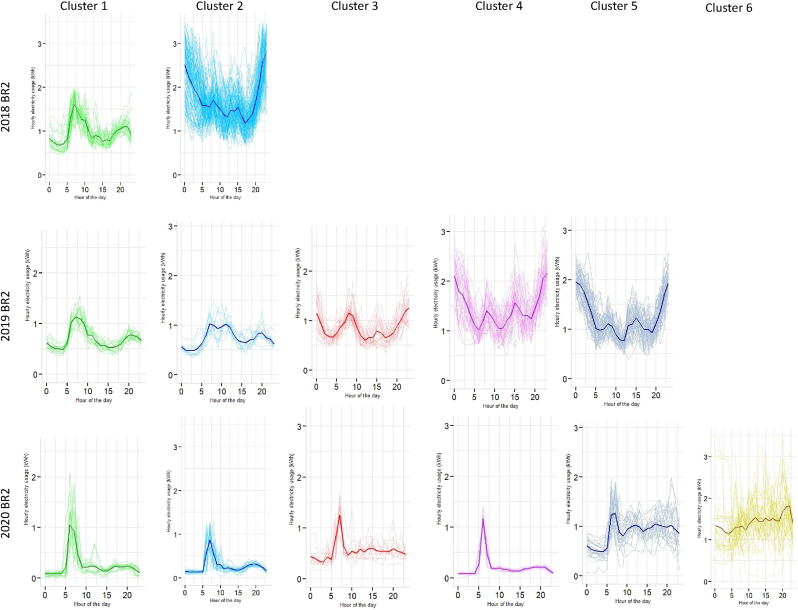
Extracted clusters of electricity demand with mean curves for two-bedroom units (BR2).

Table 1 and Fig. 7 shows the optimal model fit and the cluster membership extraction for BR2 for 2018, 2019 and 2020. Cluster 1 for all three years shows a similar trend on the daily demand curve. However, there are contrasting differences in the rest of the clusters across the time scale (see Fig. 12). Interestingly, the six clusters for 2020 show sharp peaks around 9:00 a.m. in clusters 1 to 4 (see Fig. 12 2020 BR2), whereas the same cluster number for 2019 shows a fall in demand (see Fig. 12 2019 BR2). For clusters 5 and 6 in 2020, the demand profiles are rising for the entire day with a daily mean of ∼0.87 kWh.

#### 4.2.4 Three-bedroom unit (BR3) and more-than-three-bedroom units (M3BR)

**Fig. 13:**
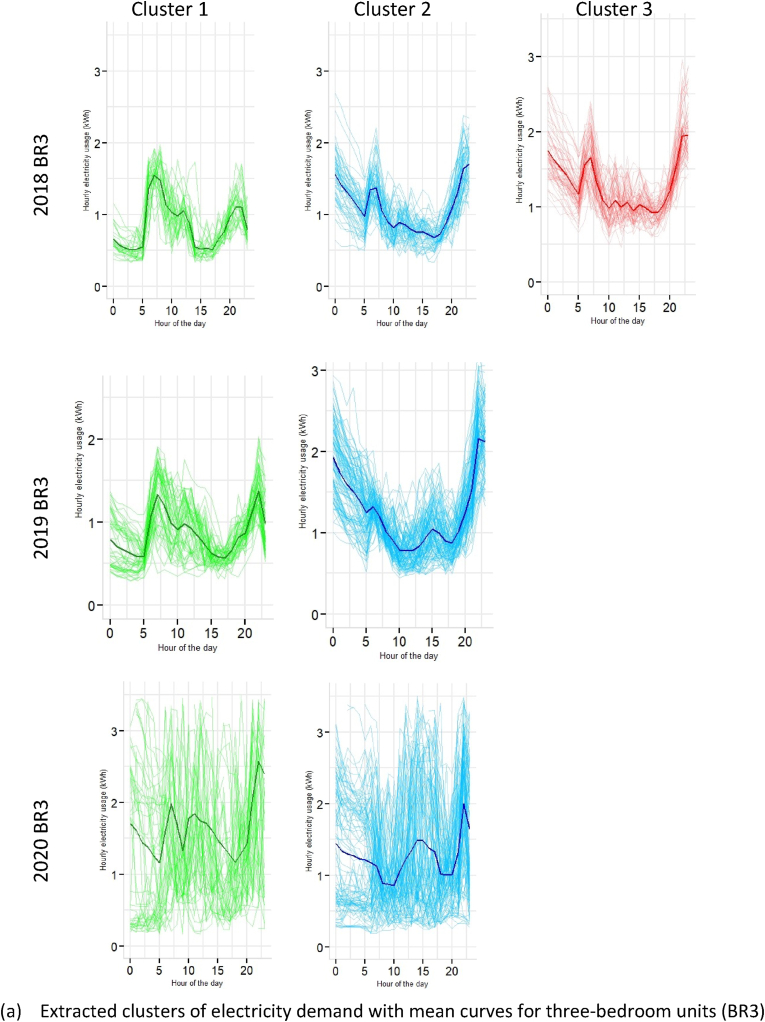

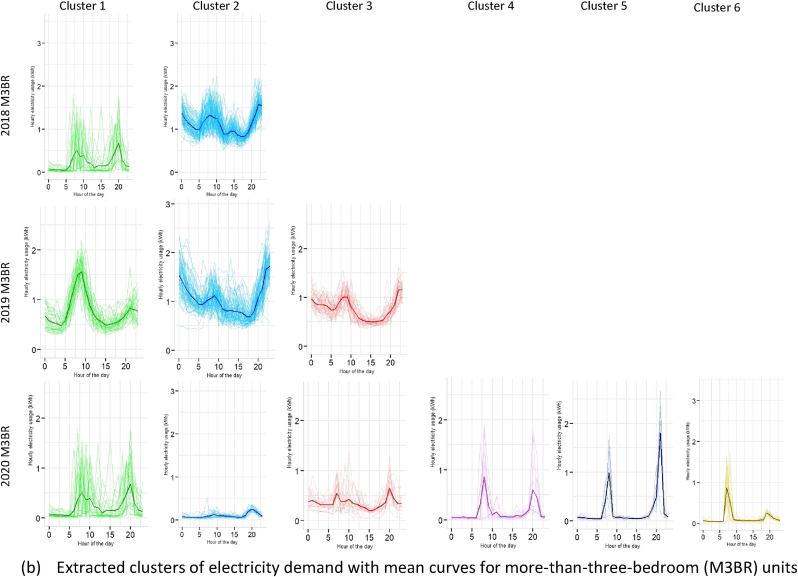
Cluster memberships for 3BR and M3BR dwelling typologies.

The cluster membership functions for BR3 and M3BR is illustrated in Table 1 and Fig. 13. It can be observed from Fig. 13a that there is a significant peak in demand for the BR3 in 2020 throughout the day across cluster 1 and cluster 2, with mean demand reaching ∼1.78 kWh and ∼1.64 kWh, respectively. Such patterns were absent for this dwelling typology in 2018 and 2019 (see Figs. 13a and 8). Similarly, the inference earlier drawn for aggregated load curves in Fig. 3 for BR3 showed a significant shift in daytime and night-time peak energy demand.

Fig. 13b illustrates the clusters for M3BR that shows a characteristic rise in demand for the 2020 case, contrary to 2018 and 2019. While both 2018 and 2019 follow similar demand patterns, clusters 1, 3, 4 and 5 in 2020 show demand spikes for 08:00–10:00 and 18:00–23:00 h in 2020 (see Fig. 13b). Typically, 3BR and M3BR are characteristic dwelling units of upper-middle and higher-income households. Therefore, the load shift in the lockdown context in these households can imply higher electricity demand for cooling, cooking and teleworking activities.

Results from section 4.1, 4.2 showed that Covid-19 related lockdowns did affect the energy consumption across dwelling typologies, especially when contextualised with the work-from-home effects. Cross-sectional investigation of the weekend and weekday energy consumption also showed that the dwelling typologies experienced different peak demand shifts during the lockdown months in 2020. The results demonstrated that RM1, 1BR and M3BR had the most variance in its temporal energy demand over the lockdown period in 2020.

## 5 Discussion

The results presented in section 4 demonstrated the effect of lockdown on electricity demand in Indian residential households compared to the demand profiles for 2019 and 2018. We evaluated the effect through an intra-and inter-dwelling unit-level analysis that revealed granular details of a daily shift in peak demands. Section 4.1 showed the aggregated results of the NILM dataset with variances in working and out-of-work hours. Fig. 5 showed that peak demand in 2020 for specific dwelling typologies increased by over 150–300%. For example, the rise in maximum peak demand for RM1 was approximately 125% during daytime and approximately 100% during night-time, over 2018 and 2019 levels. While the maximum daytime peak demand for BR1 in 2020 remained identical to 2019 and 2018 levels, we observed a night-time increase of ∼44%.

However, in a cross-sectional and aggregated analysis of the changes in weekday and weekend demand over the lockdown period, it was found that residential typologies reacted distinctly by shifts in peak demand. For example, it can be seen in Fig. 6 that the peak demand for the RM1 2020 weekday is ∼55.45% lower and ∼27.30% lower for the weekend compared to the 2018 and 2019 levels. Similarly, for BR2 in 2020, a decrease in demand was observed for weekdays (∼45.50%) and ∼9.09% for the weekend (compared to 2018 and 2019 values). In contrast, results showed ã 85% increase for weekdays and ∼55% increase for weekend consumption in 2020 for BR1 typologies (see Fig. 6).

A substantial distortion of the daily load curves was seen for 2BR units in 2020 for the night-time in Fig. 4. During the non-working hours, the maximum peak demand was ∼5.38 kWh in 2020, compared to ∼2 kWh for 2019 and 2018. The peak shifts in three-bedroom units (3BR) were even more stochastic in the entire day during the lockdown period in 2020. The maximum daily peak demand was ∼290% more than the 2019 and 2018 levels (see Section 4.1). In contrast, the aggregated demand profiles did not significantly rise in electricity demand for 3BR and M3BR typologies even during the lockdown. These typologies are characteristics of upper-middle- and higher-income households, which already have a higher energy intensity than the other dwelling types.

Section 4.2 presented a granular account of the electricity demand shift using the Gaussian Mixture Model-based clustering analysis (see section 3.3). The results were represented at an intra-dwelling unit level that specifically describes the shape of the load curves in specific clusters. Although it was found that the general trend in demand clusters for 2018 and 2019 remained similar across all dwelling typologies, there was a drastic shift in the demand profiles for 2020 (as also illustrated through bivariate density profiles in Fig. 7). These shifts were more profound for one room (RM1) (see Fig. 10), one-bedroom (BR1) (see Fig. 11) and two-bedroom (BR2) units (see Fig. 12) that typically house lower-middle and middle-income working populations.

Occupancy is never zero during work hours for low- and middle-income dwelling typologies as at least one of the household members remains in the house (Bardhan and Debnath, 2016). The load curves for 2018 and 2019 for RM1, BR1 and BR2 support this occupancy pattern (see Fig. 4). Additionally, Fig. 5 show significant variance in the work and out-of-work hours energy demand for these years, indicating a distinction between working and non-working phases. However, on analysing 2020 datasets, the clustering results for RM1, BR1 and BR2 revealed emerging peak demand at three specific periods in 2020, 09:00–15:00 h, 10:00–17:00 h and 18:00–23:00 h (see section 4.2).

Fig. 5 also showed no significant variance in the electricity demand for work and non-working hours for these dwelling typologies. It supports our initial hypothesis that peak shifts can be attributed to the work-from-home and lockdown effects. In addition, it can be due to increased usage of display devices, cooling devices, telework equipment and electric cooking in specific dwelling typologies. However, we could not extract the exact appliance profiles from the load curves as it was beyond the scope of this study.

A critical feature of this study demonstrated the role of digitalisation like smart meters and non-intrusive load monitoring in determining the shifting daily demand curves in urban India. Furthermore, with pandemic impacted changing working norms, this study shows that some dwelling typologies have experienced peak shifts that could have caused higher bills during lockdown periods. For example, post lockdown in 2020, there were several newspaper reports of over 1000% rise in electricity bills for BR2 dwelling units in Indian megacities (see [1], [2]).

## 6 Conclusion and policy implications

This study evaluated the effect of lockdown on residential electricity demand in urban India by dwelling typology. We used a novel and publicly available non-intrusive load monitoring (NILM) dataset from 13 cities across India. We used a climate normalised and a data-driven approach using unsupervised machine learning to investigate changing load curves as work-from-home demand response in specific dwelling typologies. We also show the importance of digitalisation, like NILM through smart meters, in preparing for post-pandemic energy demand and possible hybrid working scenarios.

Our results showed that during the lockdown in 2020, maximum peak load overshoot by almost 150–200% across one-room units (RM1), one-bedroom units (BR1) and two-bedroom units (BR2). The Gaussian clusters further demonstrated that while the load curves for 2018 and 2019 mirror each other in most cases, the 2020 curves are highly stochastic across the residential typologies. For example, mean daily load curves for 2020 clusters have shown peaks around 09:00–15:00, 10:00–17:00, and 18:00–23:00, which was a characteristic work-from-home effect absent in the 2018 and 2019 clusters where load peaked at 09:00–12:00 h. In addition, a cross-sectional and aggregated investigation of the weekend and weekday profile showed varied peak shits across the dwelling typologies with a general decreasing trend across RM1, BR2 and M3BR typologies. This aggregated behaviour is coherent with the existing literature.

This study utilised a publicly available non-intrusive load monitoring dataset called NEEM, which the Government of India commissioned as a pilot study. Our data-driven approach revealed significant opportunities for future digitalisation efforts, mainly focusing on data quality, reliability and accessibility. For example, no socio-demographic information was publicly provided with the NEEM database. Furthermore, a disclaimer mentioned that the surveyed households were unbalanced as per the climatic zones and income categories. Therefore, it restricted our analysis from evaluating climate-driven energy demand correlations with the housing typologies. In addition, we could not normalise the energy use data concerning the income categories due to embedded selection biases. From a data policy perspective, a key conclusion is a need for sensitivity towards the accessibility of associated meta-data. It would improve the quality of any public datasets and improve the trust and reliability of digitalisation initiatives.

Three critical policy implications can be drawn from the dependency of shifting load curves and peak demand on the residential housing typologies during the lockdown periods. First, in post-Covid hybrid work scenarios, daily load curves can shift significantly, and demand-side management may need appropriate adjustments, failing to cause an unexpected rise in electricity bills. It was observed in several Indian megacities during the first lockdown period. Second, extended work-from-home or hybrid scenarios may demand restructuring tariff mechanisms across urban India as our results showed that the number of rooms determines energy demand. Due to data restrictions, we could not establish a correlation between room size, income categories, and energy consumption in work-from-home conditions. However, we found that single room and more-than-three-bedroom dwelling units experienced the most significant variances in the energy demand. The third implication is the need for rapid smart metering and digitalisation in India to understand better factors that shape residential electricity demand. Improved data quality and reliability are critical in a digitised power system that can empower citizens, policymakers, and researchers.

The methodological approach adopted in this study is highly scalable and can be replicated in large scale analysis due to its data-centric nature. However, key learning from this paper is that socioeconomic metadata of the end-users is equally critical for deriving meaningful interpretation from large data streams. The public NEEM dataset used in this study restricted access to the socioeconomic metadata like household income range, age distribution, appliance ownership and employment type, which limited us from deriving a comprehensive understanding of the disruptive shocks (like COVID-19 lockdowns) on the residential electricity demand. Additionally, despite a broad spread of monitoring points across 13 cities and five climatic zones, this paper could not specifically utilise the embedded climatic dependencies due to a lack of access to local weather data. We further note that such climate-related metadata can help in comprehensive analysis. Nonetheless, our study provides critical data policy-related evidence in the context of digitalisation that public data infrastructure should be accompanied with contextual meta-data to enable data-centric policymaking.

Digitalisation in countries like India is in an experimental stage, and this paper provides an analytical route to leverage early-stage data infrastructure like the NEEM database. However, incomplete datasets and lack of contextualised metadata pose severe challenges handled through several assumptions that limit this paper's generalisability. For example, an assumption was made regarding the stiff working hours following the 9 to 5 work norms which is subjective to the personal and professional characteristics of the user. Similarly, we did not know how many residents worked remotely or the employment characteristics of the households. Moreover, the data resolution was set to hours by the NEEM portal, which led to disregard the unavoidable differences in the energy consumption of the installed electrical office equipment (like the number of monitors, computers, laptops, and other IT devices) and household appliances. A hertz or seconds level granularity of this consumption data could have given us more information about the appliance characteristics. The Gaussian Mixture Model based-approach presented in this paper can further enhance the detection of appliance-led demand shifts, which emphasises the future compatibility of our methodological approach.

As mentioned above, the lack of socioeconomic and local weather data of the monitored households further restricted the holistic treatment of the lockdown effects. Therefore, future policy studies using such a public dataset should emphasise its socioeconomic and climatic contextualisation, which can aid in better demand forecasting and energy management.

## CRediT authorship contribution statement

**Ramit Debnath:** Conceptualization, Methodology, Validation, Formal analysis, Investigation, Resources, Data curation, Writing – original draft, Writing – review & editing, Visualization, Supervision, Funding acquisition. **Ronita Bardhan:** Conceptualization, Methodology, Validation, Formal analysis, Writing – review & editing, Visualization, Supervision, Funding acquisition. **Ashwin Misra:** Data curation. **Tianzhen Hong:** Writing – review & editing. **Vida Rozite:** Writing – review & editing. **Michael H. Ramage:** Writing – review & editing, Funding acquisition.

## Declaration of competing interest

The authors declare that they have no known competing financial interests or personal relationships that could have appeared to influence the work reported in this paper.

## Figures and Tables

**Table A1:** Bureau of Energy Efficiency's sample specification across the five climatic zones (Source: BEE (2021))

Climatic Zone	Population Proportion	Number of Cities	Sample	Sample Percentage Allocation
Hot-Dry	17%	2	30	15%
Temperate	4%	1	20	10%
Composite	37%	5	70	34%
Warm-Humid	41%	4	70	34%
Cold	1%	1	15	7%
Total	100%	13	205	100%

**Table A2:** Bureau of Energy Efficiency's sample specification across dwelling typologies (Source: BEE (2021))

City	Sample	Census data					Sample as emergent as per dwelling types				
		1RM	1BR	2BR	3BR	M3BR	1RM	1BR	2BR	3BR	M3BR
Ahmedabad	15	36%	33%	18%	7%	4%	5.40	4.95	2.70	1.05	0.60
Bangalore	10	30%	31%	19%	8%	5%	3.00	3.10	1.90	0.80	0.50
Chandigarh	5	38%	26%	19%	9%	7%	1.90	1.30	0.95	0.45	0.35
Chennai	25	37%	32%	17%	6%	4%	9.25	8.00	4.25	1.50	1.00
Guwahati	5	24%	28%	20%	13%	13%	1.20	1.40	1.00	0.65	0.65
Hyderabad	15	33%	33%	20%	8%	4%	4.95	4.95	3.00	1.20	0.60
Indore	10	25%	32%	19%	12%	10%	2.50	3.20	1.90	1.20	1.00
Jaipur	15	26%	29%	19%	13%	11%	3.90	4.35	2.85	1.95	1.65
Kolkata	20	42%	32%	14%	6%	4%	8.40	6.40	2.80	1.20	0.80
Lucknow	10	31%	31%	17%	11%	8%	3.10	3.10	1.70	1.10	0.80
Mumbai	25	42%	28%	15%	6%	4%	10.50	7.00	3.75	1.50	1.00
New Delhi	35	32%	30%	20%	10%	6%	11.20	10.50	7.00	3.50	2.10
Shimla	10	27%	30%	17%	12%	12%	2.70	3.00	1.70	1.20	

**Table A3:** Parameterisation of the within-group covariance matrix for multidimensional data available in the mclust package and the corresponding geometric characteristics (Source: (Scrucca et al., 2016))

**Model**	$\Sigma_{k}$	**Distribution**	**Volume**	**Shape**	**Orientation**
EII	λI	Spherical	Equal	Equal	–
VII	$\lambda_{k}I$	Spherical	Variable	Equal	–
EEI	λA	Diagonal	Equal	Equal	Coordinate axes
VEI	$\lambda_{k}A$	Diagonal	Variable	Equal	Coordinate axes
EVI	$\lambda A_{k}$	Diagonal	Equal	Variable	Coordinate axes
VVI	$\lambda_{k}A_{k}$	Diagonal	Variable	Variable	Coordinate axes
EEE	$\lambda \mathrm{DA}D^{\text{T}}$	Ellipsoidal	Equal	Equal	Equal
EVE	$\lambda DA_{k}D^{\text{T}}$	Ellipsoidal	Equal	Variable	Equal
VEE	$\lambda_{k}\mathrm{DA}D^{\text{T}}$	Ellipsoidal	Variable	Equal	Equal
VVE	$\lambda_{k}DA_{k}D^{\text{T}}$	Ellipsoidal	Variable	Variable	Equal
EEV	$\lambda D_{k}AD_{k}^{\text{T}}$	Ellipsoidal	Equal	Equal	Variable
VEV	$\lambda_{k}D_{k}AD_{k}^{\text{T}}$	Ellipsoidal	Equal	Variable	Variable
EVV	$\lambda D_{k}A_{k}D_{k}^{\text{T}}$	Ellipsoidal	Variable	Equal	Variable
VVV	$\lambda_{k}D_{k}A_{k}D_{k}^{\text{T}}$	Ellipsoidal	Variable	Variable	Variable
